# Long-term cumulative physical activity associated with less cognitive decline: Evidence from a 16-year cohort study

**DOI:** 10.1016/j.tjpad.2025.100194

**Published:** 2025-04-30

**Authors:** Suhang Song, Meng Hsuan Sung, Diana Diaz, Zhuofan Lin, Allan D. Tate, Zhuo Chen, Janani Rajbhandari-Thapa, Grace Bagwell Adams, M. Mahmud Khan, Ye Shen, Lisa M. Renzi-Hammond, Yinzi Jin

**Affiliations:** a Department of Health Policy and Management, College of Public Health, University of Georgia, Athens, GA, 30602, USA; b Department of Epidemiology and Biostatistics, College of Public Health, University of Georgia, Athens, GA, 30602, USA; c Department of Global Health, School of Public Health, Peking University, Beijing, 100191, China; d School of Economics, Faculty of Humanities and Social Sciences, University of Nottingham Ningbo China, Ningbo, 315100, China; e Institute of Gerontology, College of Public Health, University of Georgia, Athens, GA, 30602, USA; f Institute of Global Health and Development, Peking University, Beijing, 100871, China

**Keywords:** Cumulative physical activity, Cognitive decline, Longitudinal study

## Abstract

**Introduction:**

Physical activity (PA) has been reported to delay cognitive decline. However, the role of long-term, cumulative PA (cPA) in cognitive decline remains unclear.

**Methods:**

This longitudinal study obtained data from Health and Retirement Study, 2004-2020. Global cognition was operationalized as the sum of memory and executive function scores on a battery of cognitive tests. cPA was operationalized as the area under the curve of the metabolic equivalent of tasks (MET) adjusted PA. Generalized linear mixed models were fitted to examine the associations between cPA and cognitive change.

**Results:**

This study included 13,450 cognitively healthy participants, with a mean follow-up duration of 11.06 (SD=4.91) years. Higher cPA was associated with delayed declines in global cognition (p<.001), memory (p<.001) and executive function (p<.001), and such protective benefits grew over the 16-year study period. Longer PA engagement was associated with progressively delayed cognitive decline.

**Conclusion:**

PA engagement over long timeframes may better maintain cognitive performance.

## Highlights

• Higher cumulative physical activity (PA) was associated with delayed cognitive decline.
• The protective benefits of cumulative PA grew over the 16-year study period, 2004-2020.
• Longer PA engagement was associated with progressively delayed cognitive decline.

## 1 Background

With a growing share of older adults in the population, it is estimated that, in the US, over 7 million adults aged 65 and older were living with dementia in 2020, and projections suggest that this number could exceed 9 million by 2030 and reach nearly 12 million by 2040 [1]. Alzheimer’s disease (AD), the most common type of dementia [2], is a leading cause of death in the US, with 1 in 3 older adults dying with AD or another dementia in 2021 [3]. Despite the significant burden of AD on individuals and healthcare systems, there are limited treatments available to prevent or slow its progression, and no curative therapies have been identified [1]. Given the lack of effective pharmacological interventions, modifying risk factors associated with dementia has become a critical area of research, clinical practice, and policy, with the potential to delay the onset of dementia and mitigate its impact on cognitive function.

Among the many modifiable risk factors that have been identified, physical activity (PA) as a health-related individual behavior is a fundamental determinant of overall health and well-being [4], with widespread benefits across the lifespan [5]. Research on PA behavior to address health problems is necessary to inform interventions that promote sustained PA, as individuals encounter significant barriers when attempting to modify complex behaviors. Current evidence shows that PA has emerged as one of the most promising protective measures against all-cause dementia [[6], [7], [8]], as well as AD [9], vascular dementia [10], and Parkinson’s disease [11]. PA has the potential to reduce dementia risks by 2 % [6,9], highlighting its considerable importance in public health initiatives aimed at reducing the burden of dementia in aging populations.

Prior to the dementia onset, a growing body of research has consistently shown that higher levels of PA are associated with better cognitive function [[12], [13], [14]], a slower rate of cognitive decline [7,[14], [15], [16], [17], [18], [19], [20], [21], [22], [23], [24], [25]], and a lower risk of cognitive impairment [22]. There are several mechanisms for the relationship between PA and cognitive function. First, PA has been shown to improve cognitive reserve, the brain’s ability to adapt and compensate in the face of changes due to age, pathology, or insult without developing cognitive impairment [14,16]. Besides, PA improves blood flow to the brain [26], reduces inflammation [[27], [28], [29]], which improves brain function [25,30], and assists in maintaining cognitive performance [25]. These mechanisms suggest that PA not only plays a critical role in sustaining cognitive health but may also have a preventive effect on cognitive decline throughout the aging process.

While some evidence suggests that increased PA may help delay cognitive decline, findings from a randomized clinical trial reported no significant improvement following a six-month PA intervention [31]. In addition, most studies that have investigated the effects of PA on cognitive function have focused on cross-sectional assessments of PA only at a single point in time, without considering the longitudinal effects of PA over the long run. A few studies have examined the potential benefits of cumulative physical activity (cPA) on better health in older age, such as a lower risk of joint pain/stiffness [32], and better health-related quality of life in adults [33]. The longitudinal effects of PA may differ from its cross-sectional effects, influenced by factors such as duration of PA, thus, capturing the cPA would provide a comprehensive understanding of how an individual’s long-term habitual patterns would affect cognitive decline. However, to the best of our knowledge, there is still a lack of robust evidence on the association of sustained, long-term engagement in PA with cognitive decline over time for older age.

Thus, this study aims to fill this gap in the literature by examining the longitudinal association between cPA and subsequent cognitive decline in cognitively healthy adults aged 50 years and older. Specifically, the aims of this study are (1) to examine whether cPA is associated with a reduction in cognitive decline over time and to explore the trends in this association, and (2) to assess whether the duration of PA engagement is associated with a slower rate of cognitive decline. By adopting a longitudinal perspective, this study would provide a more comprehensive understanding of the potential benefits of sustained PA for maintaining cognitive health in an aging population.

## 2 Methods

### 2.1 Data source and study population

Data were obtained from the Health and Retirement Study (HRS). This nationally representative longitudinal survey is fielded every two years with participants aged over 50 years. The analytic window spans from Wave 7 (2004, baseline)—the first wave with all variables of interest—through Wave 15 (2020)—the most recent wave of data available during the time of this analysis. All HRS participants gave verbal informed consent for their participation in the study; HRS data collection was approved by the Health Sciences and Behavioral Sciences Institutional Review Board at the University of Michigan. All the variables in the analysis are publicly available, and the study was approved by the Institutional Review Board of the University of Georgia (approved IRB #PROJECT00008358).

**Fig. 1:**
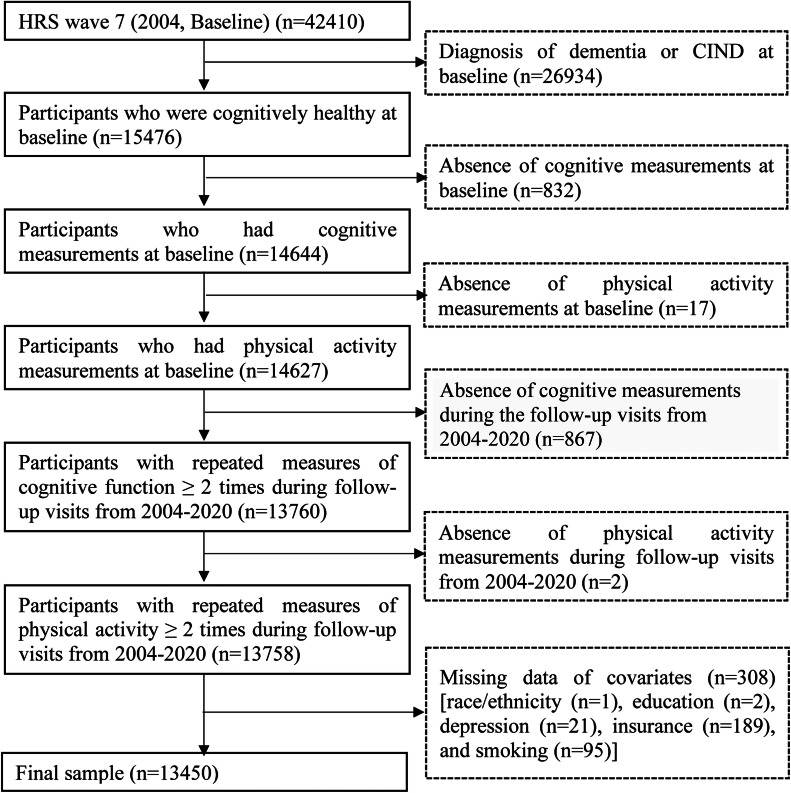
Flowchart of participant selection.

Participants were excluded if they were diagnosed as cognitively impaired but not demented (CIND) or demented at baseline, or were missing baseline PA or cognitive function measurements, or had only a single PA or cognitive function measurement in the entire study period. The participants’ selection flow chart is shown in Fig. 1. The final analytic sample includes 13,450 participants.

### 2.2 Measurement of cumulative physical activity

**Fig. 2:**
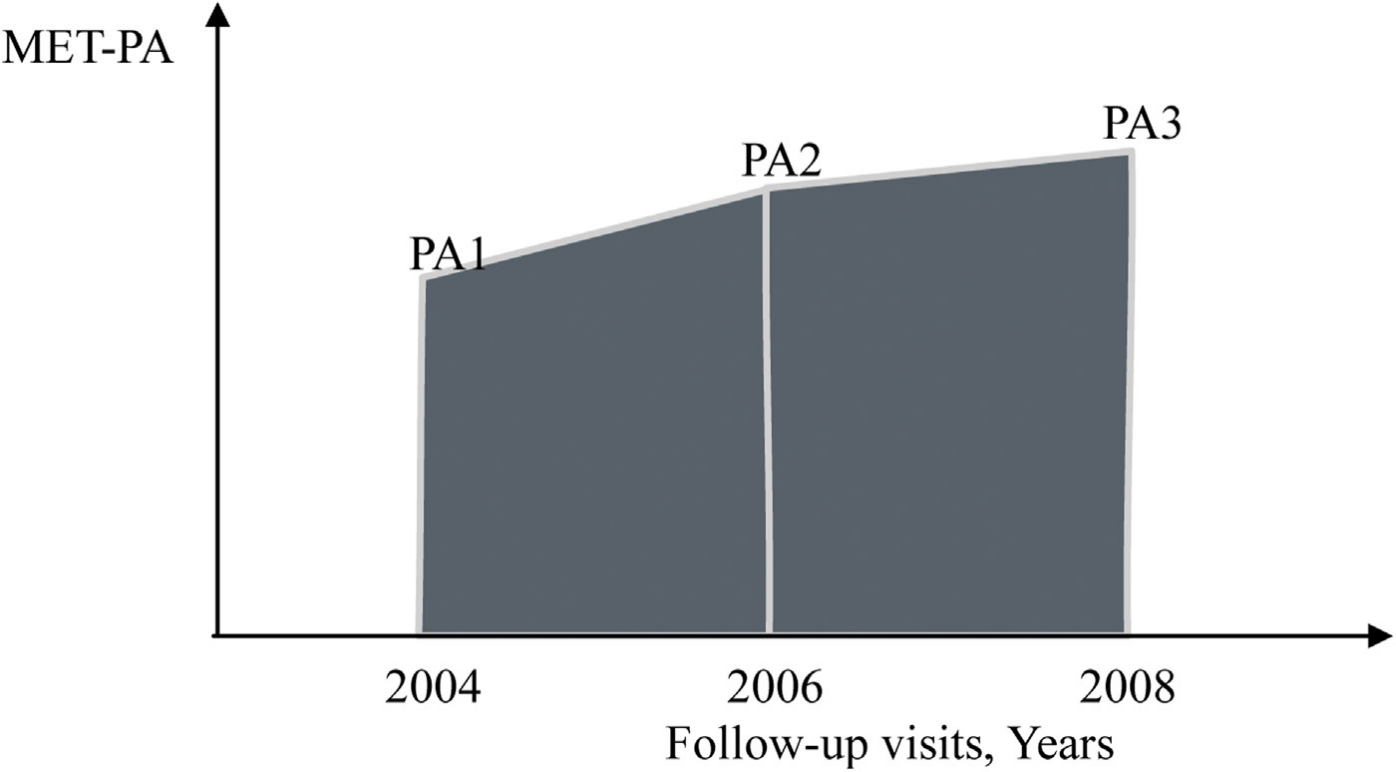
Cumulative physical activity calculation.

Abbreviations: MET = Metabolic Equivalent of Task, PA = Physical Activity.

PA was self-reported and collected via questionnaires, which demonstrated good construct, face, and predictive validity [34]. To help minimize exposure misclassification, metabolic equivalent of tasks (METs) were calculated to estimate energy expenditure based on the frequency of reported PA [35,36]. The following three questions were asked to ascertain vigorous, moderate, and light (mild) PA intensity, respectively: “How often do you take part in sports or activities that are vigorous, such as running or jogging, swimming, cycling, aerobics or gym workout, tennis, or digging with a spade or shovel?”; “And how often do you take part in sports or activities that are moderately energetic, such as gardening, cleaning the car, walking at a moderate pace, dancing, or floor or stretching exercises?”; “And how often do you take part in sports or activities that are mildly energetic, such as vacuuming, laundry, and home repairs?” Five response options were provided for each question: “hardly ever or never”, “1 to 3 times per month”; “once per week”; “greater than once per week”; or “everyday”. The five options were coded as 0, 1, 2, 3, and 4, respectively. Following a method previously described [[37], [38], [39]], individual responses to each PA question were assigned a weight (i.e., light=1.2; moderate=1.4; vigorous=1.8) in the MET calculation [40]. The weighted scores were summed across all three intensity levels of PA (range: 0-17.6), which reflected total MET-PA. cPA was calculated using the Area Under the Curve (AUC) method, and operationalized according to the trapezoid rule, which reflects the average PA accumulated over the follow-up period, on the basis of the approach adopted in previous studies [[41], [42], [43]]. For example, in Fig. 2, PA1, PA2, and PA3 denote the MET-PAs of three visits in 2004, 2006, and 2008. The cPA accumulated over two years from 2004-2006 was calculated as, according to the trapezoid rule, (PA1+PA2) × (2006-2004) / 2. The cPA accumulated over 4 years from 2004-2008 was calculated as (PA1 + PA2) × (2006 - 2004) / 2 + (PA2 + PA3) × (2008 - 2006) / 2. If the PA2 (i.e., PA in 2006) was missing, the cPA over 2004-2008 was calculated as (PA1+PA3) × (2008-2004) / 2. In this study, we performed the cPA calculation for the accumulation over 2-year, 4-year, 6-year, and 8-year periods.

### 2.3 Assessment of cognitive function

Cognitive function was measured using the validated Telephone Interview for Cognitive Status (TICS-m) [[44], [45], [46], [47]] and additionally adding web-based self-administered interview after 2018 [48]. A global cognitive score (score range: 0–27) was constructed by summing scores from memory and executive function domains[49,50]. The memory domain was evaluated via immediate (score range: 0–10) and delayed word recall (score range: 0–10) tests, and executive function was evaluated using the combined score from serial 7 subtraction (score range: 0–5) and backward counting (score range: 0-2) tests [51]. Higher scores indicated better cognitive performance [51,52]. Participants scoring between 12 and 27 were categorized as cognitively healthy, those scoring between 7 and 11 were classified as CIND, and individuals scoring between 0 and 6 were defined as demented. Due to large variations in the cognitive scores, Z-scores for each of the two cognitive domains were calculated [53]. Z-scores for global cognition were re-standardized using the mean of the two domains’ Z-scores. The means and Standard Deviations (SDs) of the baseline (i.e., 2004) sample were used in the calculation of Z-scores in all subsequent year operationalization of cognitive function Z-scores to avoid the influence of dropout (e.g., mortality selection and loss to follow-up) over the 16 years [54,55]. A cognitive Z-score of -1 at any follow-up visit indicates that the score was one SD below the mean cognitive score at baseline [42].

### 2.4 Covariates

The analysis adjusted for demographic characteristics, socioeconomic status (SES), and health features. Demographic factors include age, gender (male and female), and race/ethnicity (non-Hispanic White, non-Hispanic Black, non-Hispanic Other, and Hispanic). SES consists of educational attainment (less than high school, GED credential, high school, college, and college or above), employment status (employed, unemployed, retired, disabled, and not in the labor force), and insurance status (yes and no). Health features are comprised of depression (yes and no), smoking (never smoked, and ever/current smoker), and the number of chronic diseases (i.e., hypertension, diabetes, cancer, chronic lung disease, heart disease, and stroke).

### 2.5 Statistical analysis

Descriptive data were presented as mean ± SD. To examine the longitudinal association between cPA and cognitive decline, generalized linear mixed (GLM) models were fitted with random intercepts at both household and participant levels, as well as a random slope for the duration of follow-up time to account for differential follow-up duration across the study period. Since cPA may affect cognition in subsequent years rather than at the same time as the initial cPA measurement, this study utilized cPA in a lag structure as a predictor of an individual’s follow-up change in cognitive z-score from baseline. Lag years represent the temporal delay between the measurement of cPA and its subsequent impact on cognitive decline. cPA accumulated over a given time period (e.g., 2004–2006) was used to predict cognitive function assessed in subsequent years (e.g., 2006 onward). For example, a lag of two years indicates that cognitive decline is assessed two years after the initial measurement of cPA. This approach aligns the temporal relationship between cPA accumulation and its delayed effects on cognitive outcomes, ensuring that cPA from earlier time points is linked to later cognitive performance.

The dependent variables in the GLM models were cognitive Z-scores (i.e., global cognitive Z-score, memory Z-score, and executive functional Z-score). The independent variables in the GLM models included cPA, follow-up visit, cPA × follow-up visit, and covariates. The coefficient of the interaction term cPA × follow-up visit can reflect the association between cPA accumulated in different intervals and cognitive decline over increased induction periods, with a negative value representing accelerated cognitive decline. Huber-white robust standard errors were computed to minimize model misspecification [56,57], and the a priori type I error rate was set to the .05 level. All model fitting and data cleaning were conducted in Stata 18 MP (College Station, TX).

In order to ensure robust findings, two sensitivity analyses were performed. First, participants were excluded once they developed cognitive impairment no dementia (CIND) or dementia, thereby reducing the potential recall bias for PA associated with cognitive impairments. Second, participants with chronic diseases at baseline were excluded to isolate the influence of baseline health on the observed associations.

## 3 Results

### 3.1 Study population characteristics

At baseline, the study sample had an average age of 64.87 (SD=10.40) years, and women comprised 60.76 % of the cohort with a follow-up time of 11.06 (SD=4.91) years on average (Table 1). The mean global cognitive score at baseline was 16.95 (SD=3.03) out of 27. The sample’s racial/ethnic composition was predominantly non-Hispanic White (79.52 %). Most participants were either employed (36.79 %) or retired (51.55 %), with about half (49.48 %) of them with educational attainment of college or above. On average, participants engaged in vigorous PA one to three times per month (mean [SD]=1.07 [1.34]), and moderate PA once per week (mean [SD]=2.21 [1.20]) (Table 1).

**Table 1:** Baseline characteristics of participants (N=13,450).

Variables	Characteristics
Follow-up time (mean [SD] in years)	11.06 (4.91)
Global cognitive score (mean [SD])	16.95 (3.03)
Memory score (mean [SD])	10.97 (2.71)
Executive function score (mean [SD])	5.98 (1.34)
MET-PA (mean [SD])	8.06 (3.87)
Vigorous PA (mean [SD])	1.07 (1.34)
Moderate PA (mean [SD])	2.21 (1.20)
Light PA (mean [SD])	2.53 (0.93)
Age (mean [SD] in years)	64.87 (10.40)
Gender ( %)	
Men	5,278 (39.24 %)
Women	8,172 (60.76 %)
Race/ethnicity ( %)	
Non-Hispanic White	10,695 (79.52 %)
Non-Hispanic Black	1,456 (10.83 %)
Non-Hispanic Other	287 (2.13 %)
Hispanic	1,012 (7.52 %)
Educational attainment ( %)	
Lower Than High-school	1,884 (14.01 %)
GED	598 (4.45 %)
High-school graduate	4,313 (32.07 %)
College	3,325 (24.72 %)
College and above	3,330 (24.76 %)
Labor status ( %)	
Employed	4,948 (36.79 %)
Unemployed	228 (1.70 %)
Retired	6,934 (51.55 %)
Disabled	254 (1.89 %)
Not in labor force	1,086 (8.07 %)
Insurance status ( %)	
Uninsured	894 (6.65 %)
Insured	12,556 (93.35 %)
Smoking ( %)	
Never smoked	5,791 (43.06 %)
Ever/current smoker	7,659 (56.94 %)
Depression ( %)	
No	11,786 (87.63 %)
Yes	1,664 (12.37 %)
Number of chronic diseases (mean [SD])	1.12 (1.06)

Abbreviation: PA=Physical Activity; MET=Metabolic Equivalent of Task; GED=General Educational Development

### 3.2 Association between cPA and cognitive decline

**Fig. 3:**
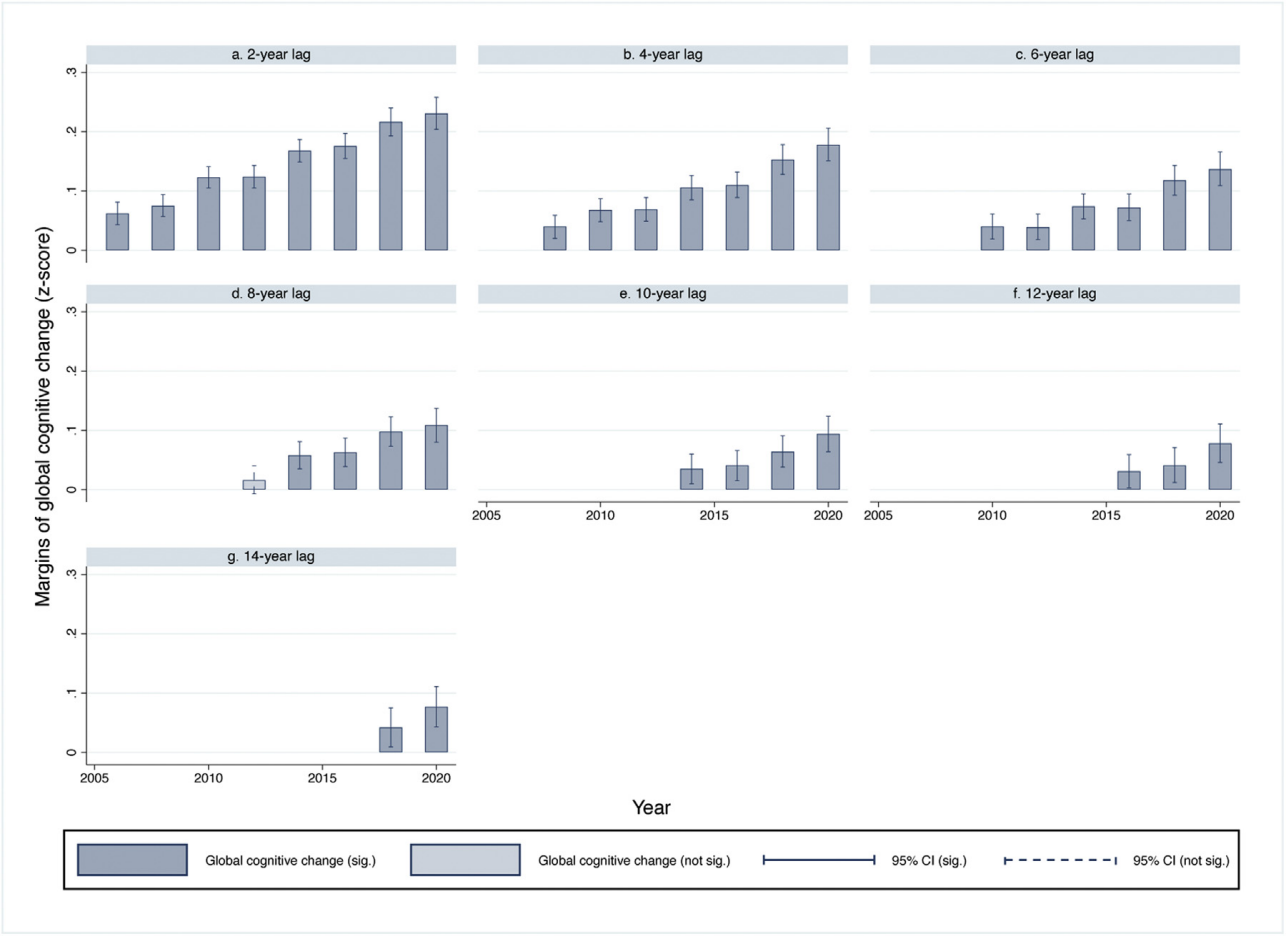
Marginal effect of cPA accumulated over two years on the subsequent global cognitive decline z-score in the following two to 14 years (2004-2020).

Abbreviations: cPA = Cumulative Physical Activity, 95 % CI = 95 % Confidence Interval. Adjusted for age, gender, race/ethnicity, educational attainment, insurance status, labor status, depression, smoking, and number of chronic diseases.

Higher cPA was associated with delayed global cognitive decline, with protective benefits progressively intensifying over the 16-year study period (p<.001) (Fig. 3). Specifically, at the first follow-up visit in 2006 (two years from baseline), one SD increase in cPA accumulated over two years corresponded to a 0.062 smaller decline in global cognitive z-score (95 % confidence interval [CI]=0.043, 0.081, p<.001) relative to baseline levels in 2004. By the eighth follow-up visit in 2020 (16 years from baseline), one SD-unit increment in cPA was linked to 0.231 smaller decline in global cognition (95 % CI=0.204, 0.258, p<.001), nearly four times the magnitude of the association observed in 2006 (Fig. 3). Practically, a one SD increase in cPA suggests an improvement from engaging in vigorous PA one to three times per month to once per week, and from moderate PA once per week to more than once per week. This pattern suggests a potential buffering effect of cPA on cognitive aging (Fig. 3). Similar increasing trends in the protective effect of cPA were evident on global cognitive decline in the subsequent four years, extending up to 14 years after the initial cPA accumulation period (Fig. 3), indicating that the protective impact of sustained PA continued to strengthen over time. For specific cognitive domains, higher cPA was also associated with mitigating memory decline across the study period, with progressively larger protective effects emerging in later years (Appendix Fig. 1). A similar pattern was exhibited for executive function, although the impact of cPA diminished when considering longer observation periods (Appendix Fig. 2). Likewise, examining cPA accumulated over four-, six-, and eight-year durations revealed consistent protective associations with subsequent declines in global cognitive function, memory, and executive function (Appendix Fig. 3-5).

In the sensitivity analyses, consistent findings were observed in the samples excluding CIND/demented participants in the follow up visits, and participants with chronic diseases at baseline (Appendix Fig. 6-7).

### 3.3 Association between length of PA engagement and cognitive decline

**Fig. 4:**
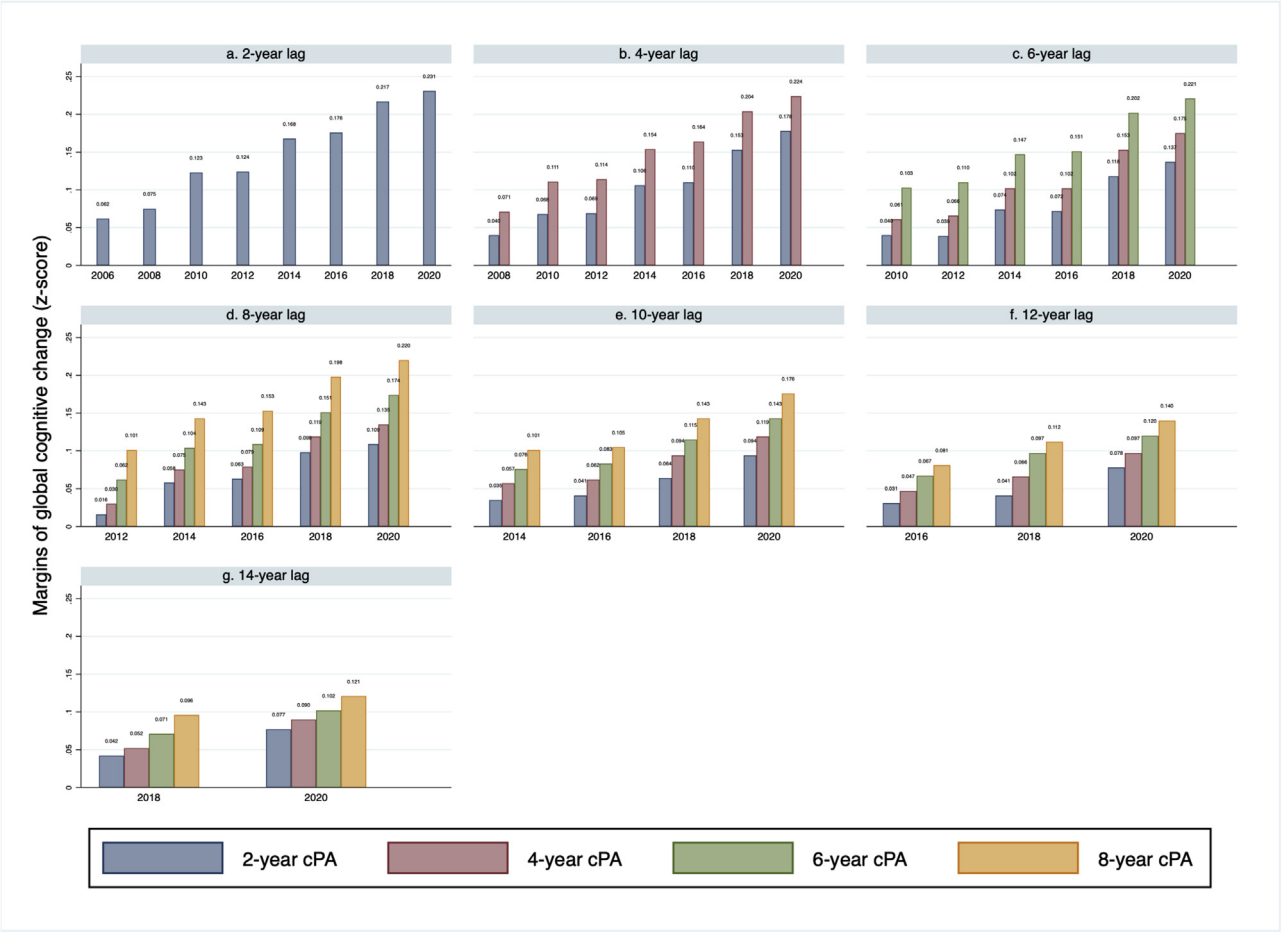
Marginal effect of cPA accumulated over different years on the subsequent global cognitive decline z-score for each follow-up visit (2004-2020).

Abbreviations: cPA = Cumulative Physical Activity. Adjusted for age, gender, race/ethnicity, educational attainment, insurance status, labor status, depression, smoking, and number of chronic diseases.

Fig. 4 illustrates that prolonged engagement in PA was associated with progressively greater protection against global cognitive decline. The x-axis denotes the follow-up years. For instance, in panel e, a one-SD increase in cPA accumulated over two years from 2004 to 2006 was associated with a 0.035 smaller decline in global cognitive z-score by 2014 (95 % CI=0.010, 0.060, p=.006) (Fig. 4). Examining successively longer durations, SD-unit increases in cPA over four years (2004-2008), six years (2004-2010), and eight years (2004-2012) were linked to incrementally larger effects in global cognitive z-score. Specifically, cPA was linked to a 0.057 smaller decline in global cognition over four years (95 % CI: 0.032–0.083; p<.001), a 0.076 smaller decline over six years (95 % CI: 0.050–0.102; p<.001), and a 0.101 smaller decline over eight years (95 % CI: 0.075–0.127; p<.001) (Fig. 4). Similar patterns emerged for both memory (Appendix Fig. 8) and executive function (Appendix Fig. 9).

## 4 Discussion

Based on data from the HRS cohort of 13,450 cognitively healthy adults aged 50 years and older, our study innovatively demonstrated that longitudinal cPA served as a significant protective factor in slowing cognitive decline, and the benefits grew over the 16-year study period. Besides, extending the duration of PA was associated with progressively delayed cognitive decline. However, less than one in four adults in the US engage in sufficient PA to reap optimal health benefits [58], therefore, effective promotion strategies are warranted. Theories of behavior change are essential to understand cPA and provide an organizing framework for effective intervention.

The prolonged benefits of cPA over the study period are particularly meaningful when one considers the nature of age-related cognitive decline. In healthy adults, age-related cognitive decline is statistically normal (i.e., most people experience it) but can progress to a prodromal (pre-clinical) stage, which can progress to mild cognitive impairment [59]. Approximately 15 % of older people with mild cognitive impairment receive a diagnosis of Alzheimer’s disease within a year [60]. Age-related cognitive decline is often progressive; consequently, strategies to prevent the earliest pre-clinical changes may delay the onset of Alzheimer’s disease by years, or for some people, indefinitely [61].

How, then, might cPA impact brain health and, consequently, improve cognitive function [14,[22], [23], [24], [25],^62^]? A growing body of literature suggests that PA promotes functional connectivity and improves neural plasticity in ways that may directly address common, negative age-related physiological changes, such as brain volume loss, loss of white matter integrity, and age-related changes in network connectivity [[63], [64], [65], [66]]. For example, animal models suggest that PA can promote neuroplasticity and enhance synaptic connectivity [67], and human research studies support these findings, suggesting that older adults who are physically active have better cerebral blood flow, better functional connectivity, and less volume loss [68,69]. Mechanistically, PA in older age is associated with elevated neurotrophic factors (e.g., brain-derived neurotrophic factor [BDNF], glial cell line-derived neurotrophic factor [GDNF], insulin-like growth factor-1 [IGF-1], vascular endothelial growth factor [VEGF] and nerve growth factor [NGF]) [[70], [71], [72]], contributing to improved cognitive performance. These growth factors promote functional connectivity and improve white matter integrity in key functional networks, such as the default mode network, parahippocampal gyri, and middle temporal gyri – key functional networks that decline in dementing illnesses, such as Alzheimer’s disease [72].

In addition to promoting white matter integrity and functional connectivity, PA may act as a proxy of cognitive reserve [[73], [74], [75], [76], [77], [78]], which is the brain’s ability to maintain cognitive function despite neural loss [74]. Sustained PA may reduce the negative impact of adverse brain markers (e.g., white matter hyperintensities) on cognitive function, thereby delaying cognitive decline [14,25]. Therefore, the full protective potential effects of PA may be realized when individuals are followed over extended periods, as they age and might experience more significant cognitive changes [79]. The growth of the cPA benefits over the course of this study underscores the importance of promoting timely and continuous PA participation among older adults to delay the acceleration of cognitive decline.

This study also demonstrates that the cPA’s protective effects on cognitive decline became more pronounced, as the duration of cPA increased, suggesting that long-term sustainable PA regimens could offer an effective, non-pharmacological strategy for preserving cognitive function and mitigating the cognitive declines typically associated with aging. In the context of future research investigating PA behaviors, whether related to the initiation of a new exercise program or the maintenance of existing PA habits, it may be advantageous to develop effective interventions that encourage individuals to reflect on the reasons they identify as important for exercising. These reasons may be translated into motivations for PA engagement, and then be examined and utilized in terms of their contributions to their overall long-term exercise behaviors.

These findings have important implications in promoting cognitive health among aging populations. First, our research highlights the importance of integrating PA as a core component of dementia prevention programs. Incorporating long-term PA into routine cognitive health assessments for older adults is essential, not only to delay cognitive decline, but also to assist in identifying individuals at higher risk of cognitive impairment and/or dementias. Public health initiatives aimed at reducing the incidence of dementia may emphasize continuous PA as a proactive measure, thereby fostering a culture of early intervention and prevention that moves beyond reactive care strategies to actively promote brain health. Importantly, even modest increases in PA frequencies, such as progressing from once per week to more than once per week for moderate PA, or from one to three times per month to once per week for vigorous PA, could play a meaningful role in delaying cognitive decline. Second, the findings could offer support in informing the design of targeted interventions that support timely, consistent, and long-term PA among older adults. Specifically, healthcare providers, community organizations, and decision-makers could collaborate to develop evidence-based guidelines detailing the recommended types, intensities, and durations of PA most likely to preserve cognitive function. Leveraging digital tools (e.g., wearables, telehealth platforms) and community-based programs could further strengthen adherence by providing ongoing support, motivation, and monitoring. Through these practical approaches, the protective effects of prolonged PA engagement can be translated into scalable, real-world strategies that help individuals maintain cognitive vitality and reduce their risk of dementia.

Although the current study provides important insights, it is not without limitations. First, the cognitive outcomes were measured using the TICS-m. While TICS-m is validated for large-scale research, it lacks the depth and granularity to capture nuances within specific functional domains. Consequently, the nonsignificant findings for executive function in later follow-up visits may partly reflect instrument-related constraints. Second, HRS relies on self-reported data, which may be subject to recall bias and social desirability bias, especially in a sample that may include participants with cognitive impairments, where self-report accuracy might be further compromised. Such misclassification could potentially bias our estimates of cPA and attenuate the observed associations with cognitive decline. Third, the HRS questionnaire does not specify an exact recall period for PA, which may also introduce misclassification bias in our cumulative PA estimates. Last, the presence of missing data and unmeasured cohort effects may limit the generalizability of these findings to future populations. For instance, emerging conditions such as long COVID might accelerate cognitive deterioration in future aging cohorts, requiring cautious interpretation of these findings in younger or different cohorts. Future research should address these limitations by employing in-depth cognitive batteries, integrating objective measures of physical and cognitive health, and examining the influence of novel health conditions on cognitive trajectories.

Despite these limitations, the study has several notable strengths. In particular, this study is among the first to assess the longitudinal effects of cPA on delaying cognitive decline over time. By capturing timely and sustained PA, cPA provides a more comprehensive perspective on how long-term engagement in PA supports cognitive health. Besides, this study serves as a first step in identifying the optimal duration of PA engagement required to prevent cognitive dysfunction, providing a basis for future research into the ideal duration of PA that may best support cognitive vitality. Furthermore, our temporal modeling approach enabled stronger capability in addressing reverse causation and detecting robust associations between cPA and subsequent cognitive decline.

## 5 Conclusion

cPA was associated with delayed cognitive decline in cognitively healthy adults aged ≥50 years, and these protective effects increased over the course of the study. Maintaining long-term PA may help better preserve cognitive performance in later life.

## Funding sources

This study was supported by Bill & Melinda Gates Foundation (INV-0045085), Beijing Nova Program (20230484284), and the National Center for Advancing Translational Sciences of the National Institutes of Health under Award Number UL1TR002378. The content is solely the responsibility of the authors and does not necessarily represent the official views of the National Institutes of Health.

## CRediT authorship contribution statement

**Suhang Song:** Writing – review & editing, Writing – original draft, Visualization, Supervision, Project administration, Methodology, Investigation, Funding acquisition, Formal analysis, Data curation, Conceptualization. **Meng Hsuan Sung:** Writing – review & editing, Data curation. **Diana Diaz:** Writing – review & editing, Formal analysis, Data curation. **Zhuofan Lin:** Writing – original draft. **Allan D. Tate:** Writing – review & editing, Methodology, Formal analysis, Data curation. **Zhuo Chen:** Writing – review & editing. **Janani Rajbhandari-Thapa:** Writing – review & editing. **Grace Bagwell Adams:** Writing – review & editing. **M. Mahmud Khan:** Writing – review & editing. **Ye Shen:** Writing – review & editing. **Lisa M. Renzi-Hammond:** Writing – review & editing. **Yinzi Jin:** Writing – review & editing, Writing – original draft, Supervision, Project administration, Funding acquisition, Conceptualization.

## Declaration of competing interest

The authors declare the following financial interests/personal relationships which may be considered as potential competing interests: Yinzi Jin reports article publishing charges was provided by Bill & Melinda Gates Medical Research Institute. Suhang Song reports article publishing charges and travel were provided by National Center for Advancing Translational Sciences. If there are other authors, they declare that they have no known competing financial interests or personal relationships that could have appeared to influence the work reported in this paper.

## Appendix Supplementary materials

Image, application 1
