# Supplementary material for: Long-term cumulative physical activity associated with less cognitive decline: Evidence from a 16-year cohort study
Source: J Prev Alzheimers Dis. 2025 Apr 30;12(6):100194. doi: 10.1016/j.tjpad.2025.100194 (PMC12434247; doi:10.1016/j.tjpad.2025.100194)
Supplement: Supplementary file 1 [file mmc1.docx]

Appendix Figure 1. Marginal effect of cPA accumulated over two years on the subsequent memory decline z-score for each follow-up visit (2004-2020)


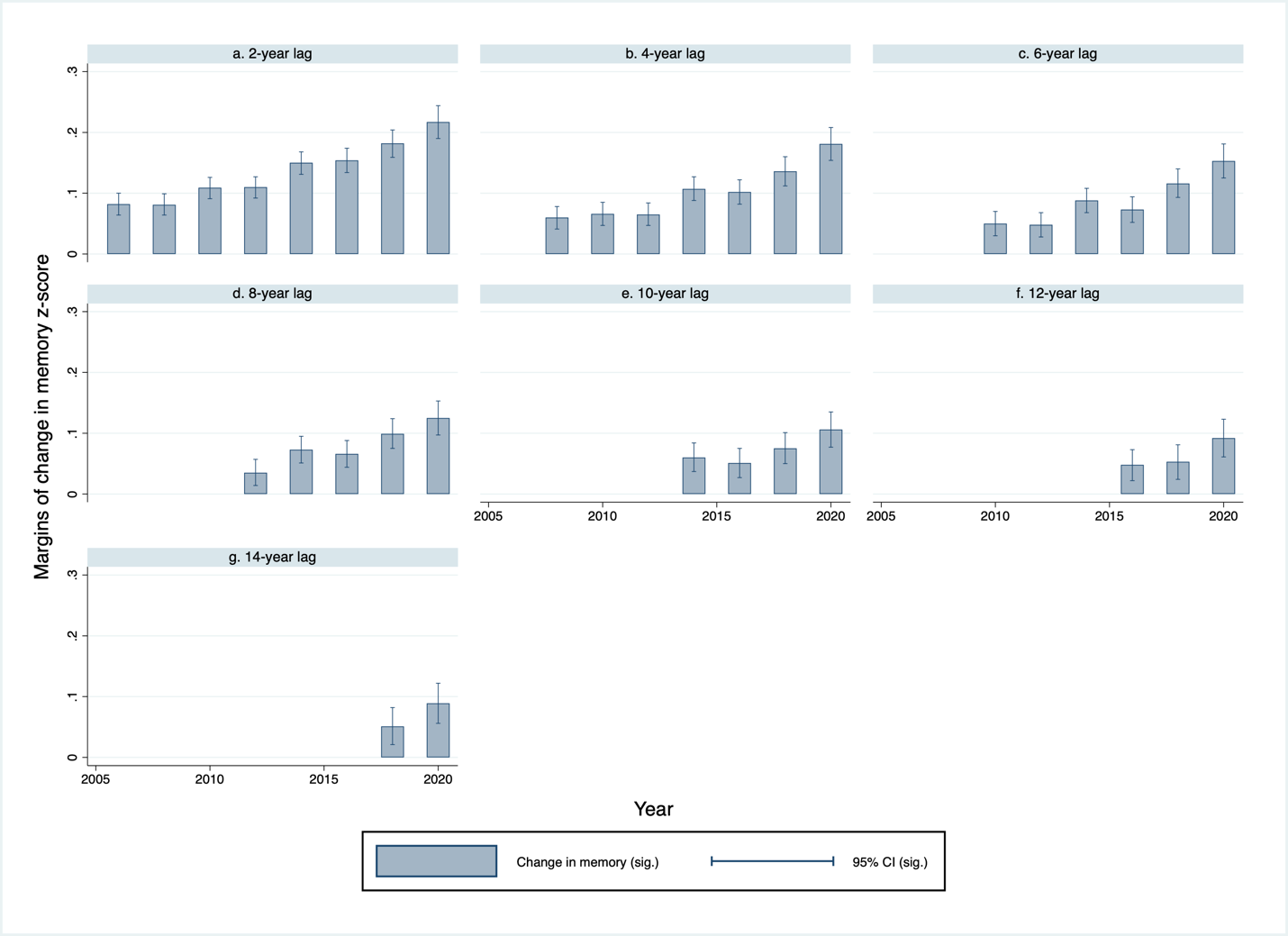
 Abbreviations: cPA = Cumulative Physical Activity, 95% CI = 95% Confidence Interval.

Adjusted for age, gender, race/ethnicity, education level, insurance status, labor status, depression, smoking, and number of chronic diseases.

Appendix Figure 2. Marginal effect of cPA accumulated over two years on the subsequent decline in executive function z-score for each follow-up visit (2004-2020)


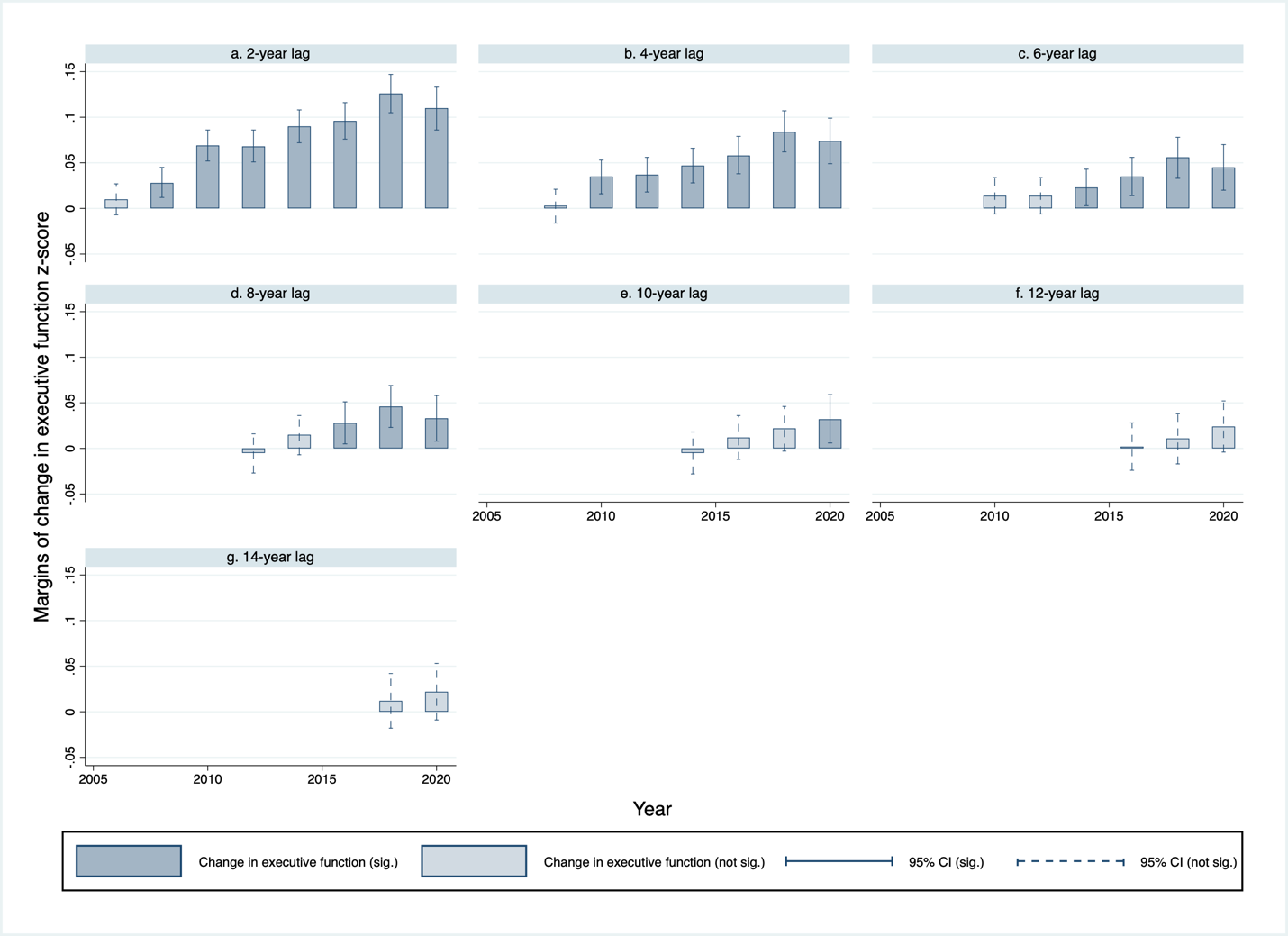
 Abbreviations: cPA = Cumulative Physical Activity, 95% CI = 95% Confidence Interval.

Adjusted for age, gender, race/ethnicity, education level, insurance status, labor status, depression, smoking, and number of chronic diseases.

Appendix Figure 3. Association between cPA accumulated over four years and the subsequent declines in global cognition, memory, and executive function for each follow-up visit (2004-2020)


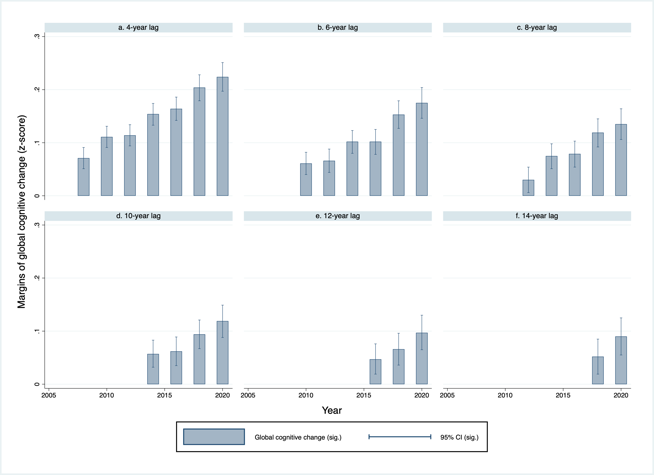

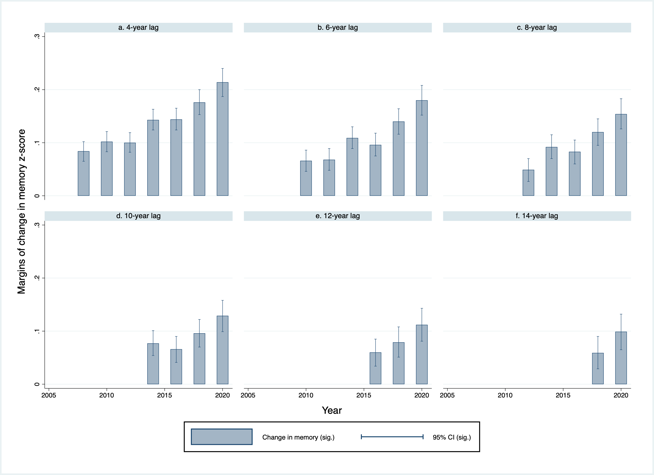

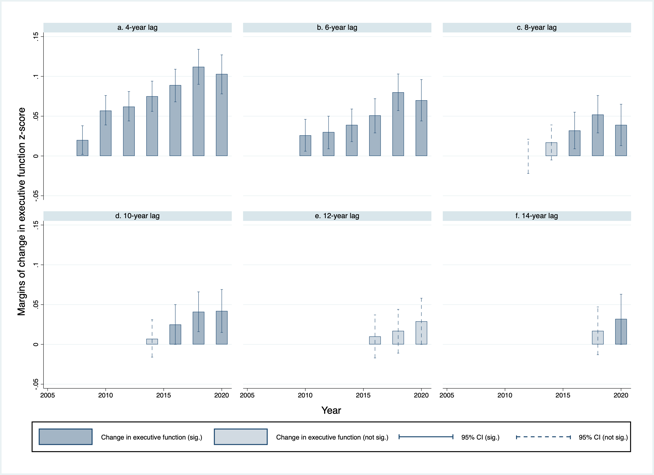
 Abbreviations: cPA = Cumulative Physical Activity, 95% CI = 95% Confidence Interval.

Adjusted for age, gender, race/ethnicity, education level, insurance status, labor status, depression, smoking, and number of chronic diseases.

Appendix Figure 4. Association between cPA accumulated over six years and the subsequent declines in global cognition, memory, and executive function for each follow-up visit (2004-2020)


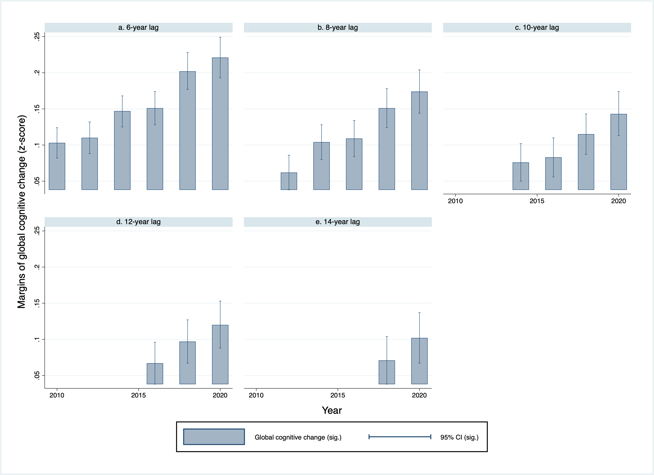

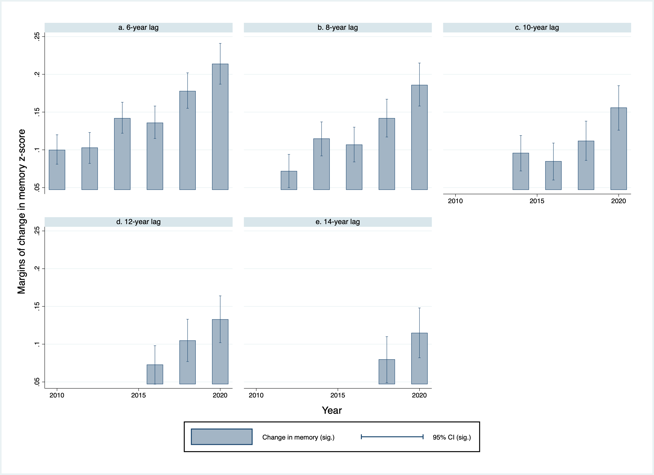

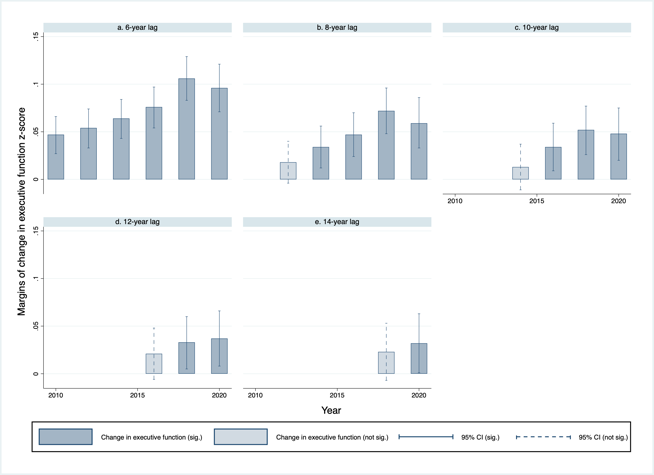
 Abbreviations: cPA = Cumulative Physical Activity, 95% CI = 95% Confidence Interval.

Adjusted for age, gender, race/ethnicity, education level, insurance status, labor status, depression, smoking, and number of chronic diseases.

Appendix Figure 5. Association between cPA accumulated over eight years and the subsequent declines in global cognition, memory, and executive function for each follow-up visit (2004-2020)


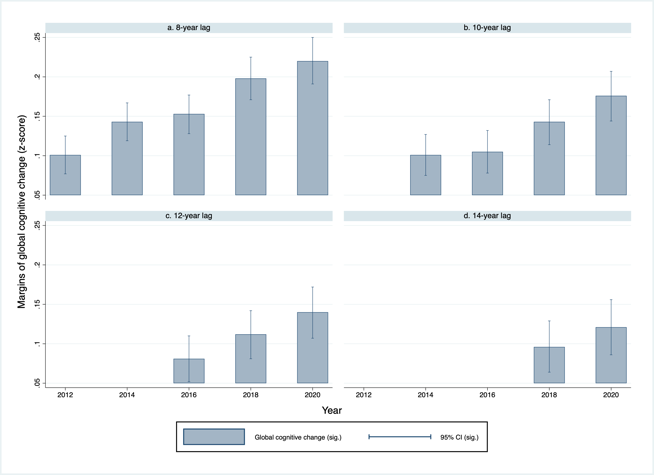

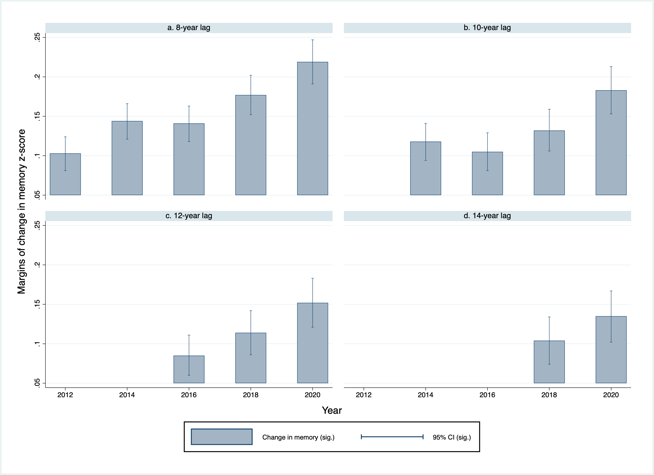

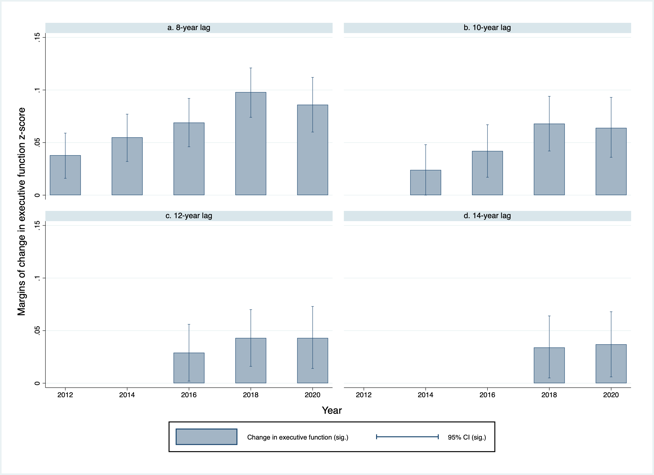
 Abbreviations: cPA = Cumulative Physical Activity, 95% CI = 95% Confidence Interval.

Adjusted for age, gender, race/ethnicity, education level, insurance status, labor status, depression, smoking, and number of chronic diseases.

Appendix Figure 6. Marginal effect of cPA accumulated over two years on the subsequent decline in executive function z-score for each follow-up visit (2004-2020), after excluding participants with CIND or dementia in the follow up visits.


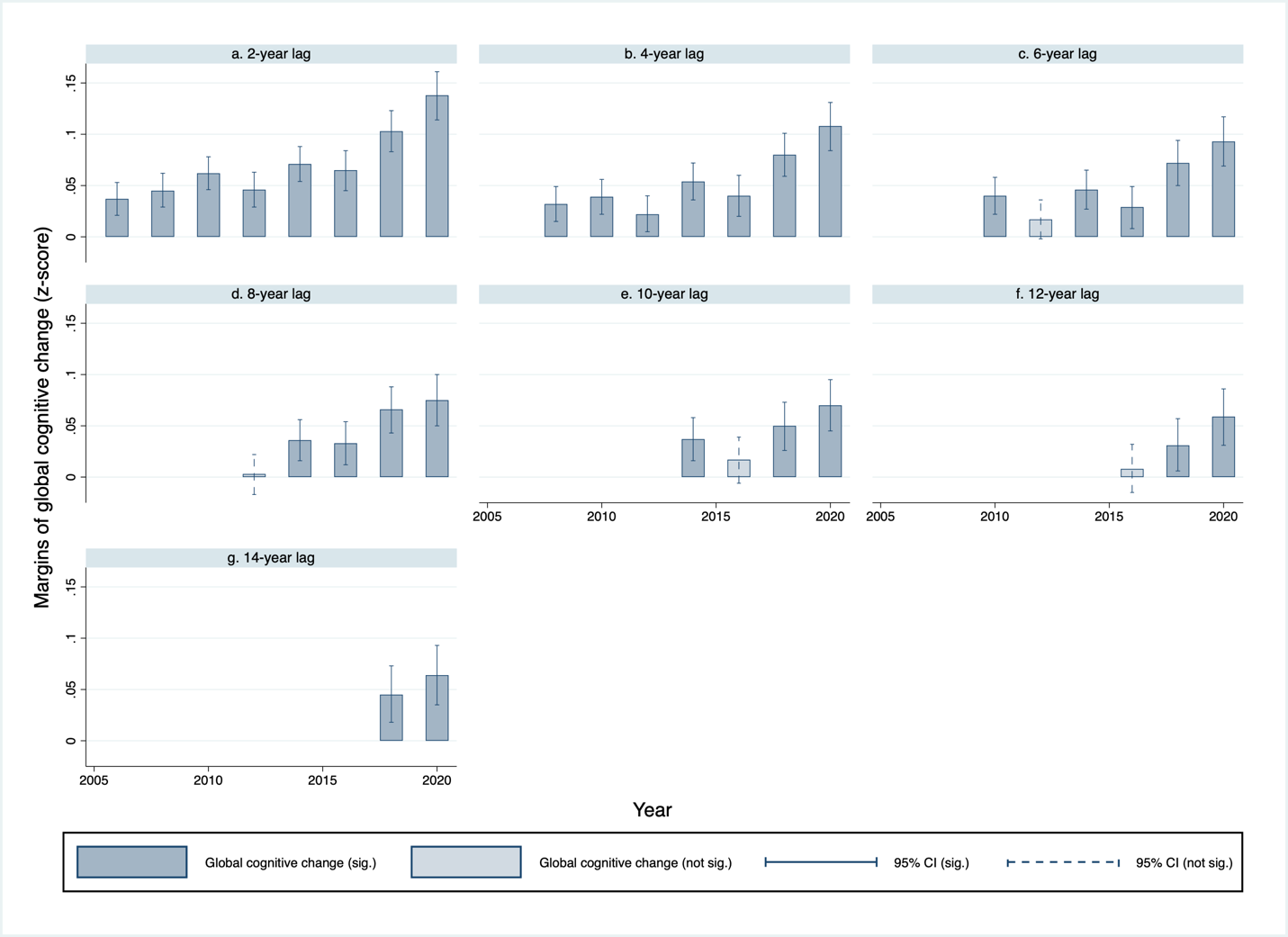


Abbreviations: cPA = Cumulative Physical Activity, 95% CI = 95% Confidence Interval.

Adjusted for age, gender, race/ethnicity, education level, insurance status, labor status, depression, smoking, and number of chronic diseases.

Appendix Figure 7. Marginal effect of cPA accumulated over two years on the subsequent decline in executive function z-score for each follow-up visit (2004-2020), after excluding participants with chronic disease at baseline.


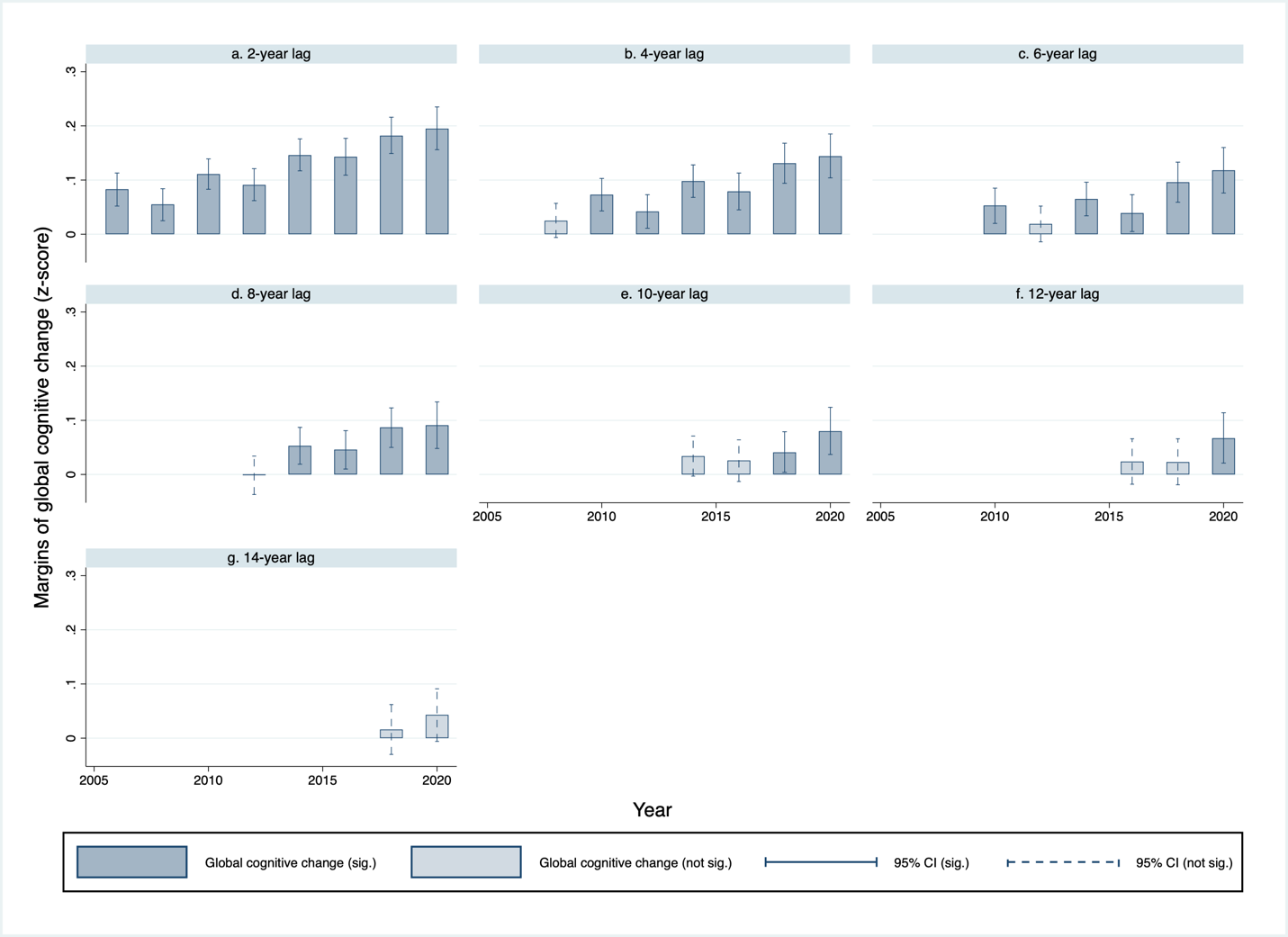


Abbreviations: cPA = Cumulative Physical Activity, 95% CI = 95% Confidence Interval.

Adjusted for age, gender, race/ethnicity, education level, insurance status, labor status, depression, smoking, and number of chronic diseases.

Appendix Figure 8. Marginal effect of cPA accumulated over different years on the subsequent memory decline z-score for each follow-up visit (2004-2020)


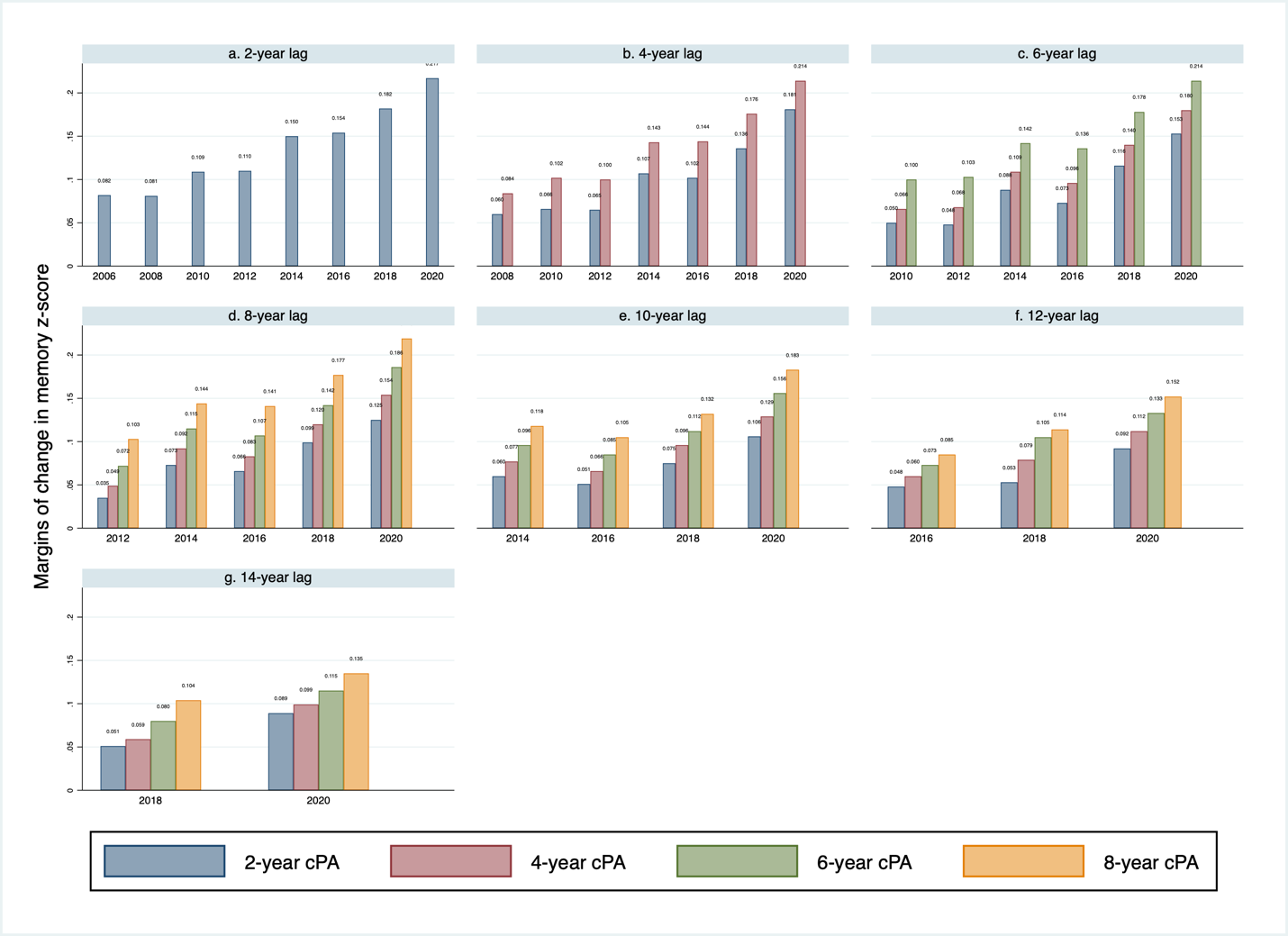
 Abbreviations: cPA = Cumulative Physical Activity.

Adjusted for age, gender, race/ethnicity, education level, insurance status, labor status, depression, smoking, and number of chronic diseases.

Appendix Figure 9. Marginal effect of cPA accumulated over different years on the subsequent decline in executive function z-score for each follow-up visit (2004-2020)


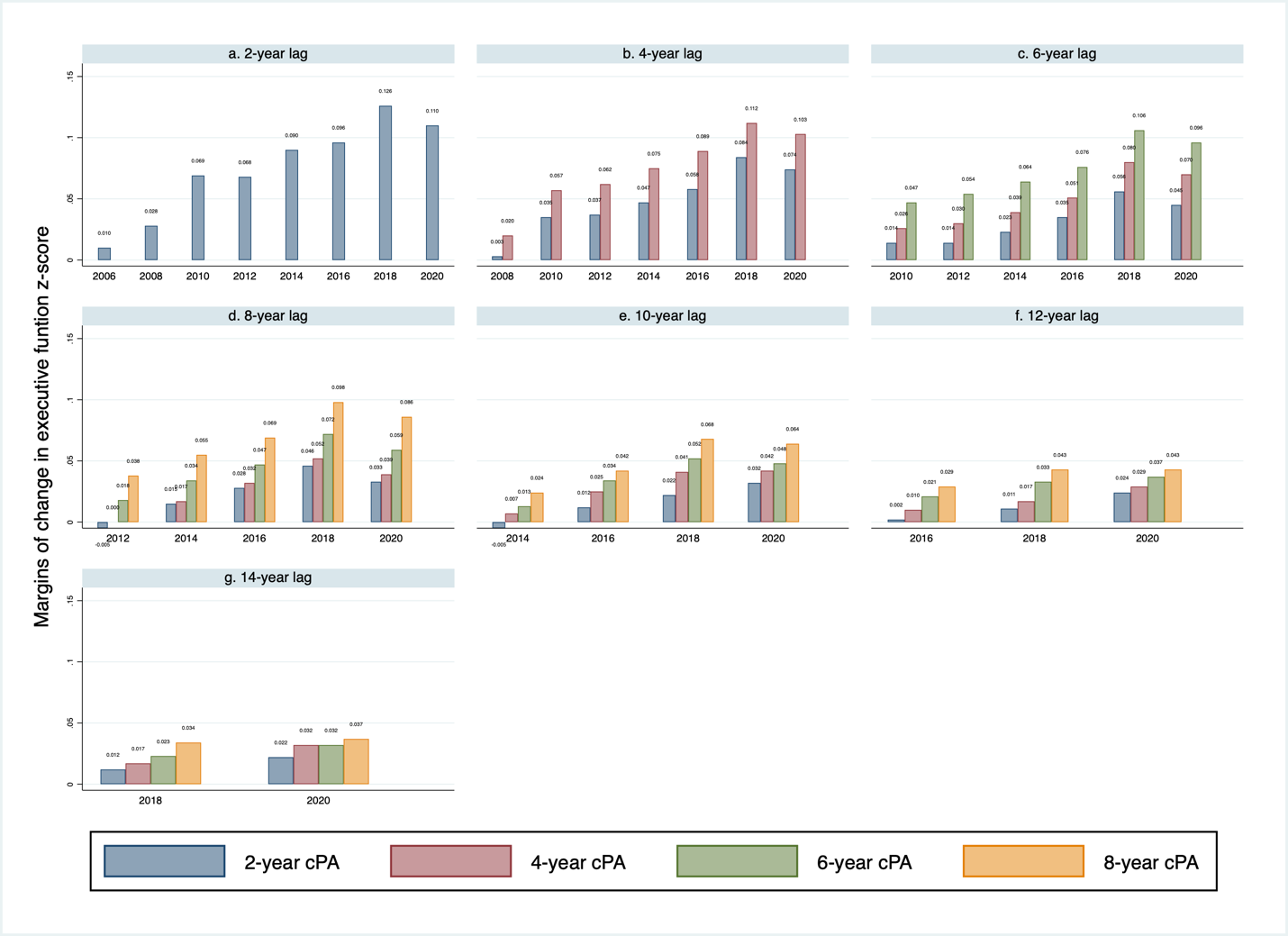
 Abbreviations: cPA = Cumulative Physical Activity.

Adjusted for age, gender, race/ethnicity, education level, insurance status, labor status, depression, smoking, and number of chronic diseases.
